# Debate on HES safety is important, but must be based on facts

**DOI:** 10.1186/1757-7241-21-66

**Published:** 2013-08-30

**Authors:** Nicolai Haase, Rasmus Müller, Anders Perner

**Affiliations:** 1 Department of Intensive Care, Copenhagen University Hospital – Rigshospitalet, DK-2100, Copenhagen, Denmark

**Keywords:** Hydroxyethyl starch, Sepsis, Critical illness, Fluid, Resuscitation

## Abstract

The Scandinavian Starch for Severe Sepsis / Septic Shock (6S) trial showed that hydroxyethyl starch was harmful compared to Ringer’s acetate in patients with severe sepsis when used according to clinical practice and in alignment with the recommendations by the manufactures and authorities. The different interpretation by Chapell and Jacob’s rely on misreading of the trial publication and is not supported by the trial data. Several hypotheses may be made regarding less harmful ways of using HES in critically ill patients, but clinicians, guideline committee members and authorities need to acknowledge that such safer ways have not yet been identified.

## Letters to the Editor

In a recent commentary in the Scandinavian Journal of Trauma, Resuscitation and Emergency Medicine Chappell and Jacob provide their view of the three largest, randomised clinical trials of hydroxyethyl starch in critically ill patients [1]. The authors’ interpretation of these trials and their view of the safety with HES are far from those of independent authors conducting high-quality systematic reviews [2-7], the US Food and Drug Administration [8] and the European Medicines Agency’s Pharmacovigilance Risk Assessment Committee [9].

Unfortunately, several claims regarding the Scandinavian Starch for Severe Sepsis/Septic Shock (6S) trial rely on misreading of the main publication of this trial [10] and are not supported by the trial data.

The 6S trial aimed at testing the effect of HES versus Ringer’s acetate as used in clinical practice in a high number of different intensive care units. The 24 hour window from diagnosis of severe sepsis resembles clinical practice, where fluid resuscitation in septic patients is initiated without waiting for the results of new blood samples to confirm the diagnosis of severe sepsis.

Forty-nine percent, not 60%, received colloids in the 24 hours prior to randomisation, but the clinician judged that fluid resuscitation was still needed as this was an inclusion criterion. Such judgement is complex, but may have been based on poor peripheral perfusion, oliguria, increased vasopressor need etc. Thus, the claim that the patients were hemodynamically stabilized at randomisation is unjustified. Also, CVP and ScvO_2_ were only registered in a minority of patients and either value may have been abnormal or considered abnormal in combination with other parameters by the clinicians assessing these patients. Neither we nor Chappell and Jacob can know how individual clinicians judged patients.

Most trial fluid was given in the first 38 hours (day 1 lasted median 14 hours) and the cumulative total dose of HES was less than the labelled maximum daily dose of HES (Table 1). Thus, the statement that HES was given in high amounts and over a prolonged period of time is not true for the majority of patients.

**Table 1:** Doses of trial fluid

	**HES 130/0.42 (N = 398)**	**Ringer’s acetate (N = 400)**	
	**Volume median (IQR)**	**Volume median (IQR)**	**P Value**
**Trial Fluid, ml**			
Day 1	1500 (1000–1500)	1500 (1000–1550)	
Day 2	1000 (300–1500)	1000 (500–1500)	
Day 3	500 (0–1000)	425 (0–1000)	
Day 4	0 (0–500)	0 (0–500)	
Day 5	0 (0–500)	0 (0–500)	
**Cumulative Dose (day 1–89)**			
In ml	3000 (1500–5000)	3000 (1800–5500)	0.20
In ml/kg	44 (24–75)	47 (25–76)	0.18

The table show volume of trial fluid according to intervention group and days after randomisation. The presentation differs from that of the primary publication of the 6S trial, but reflects exactly the same data.

The P value is from Wilcoxon rank-sum test comparing the total trial fluid volume in all patients. IQR denotes interquartile range.

During the trial, 38 of 400 patients (10%) in the Ringer’s group received synthetic colloid (almost exclusively HES). Sixty-five patients (16%) in the Ringer’s group received albumin for other indications than volume expansion. Overall, 92 (23%) patients in the Ringer’s group received a colloid during the trial, and not 32% as stated by Chappell and Jacob. As the use of albumin was similar in both intervention groups, this unlikely affected the trial results.

Most patients discontinued trial fluid due to severe bleeding or renal replacement therapy (RRT) as stipulated in the protocol [11]. To exclude these patients from the primary analysis would mask the harmful side effects of HES. However, per-protocol analyses excluding patients, who discontinued trial fluid for other reasons, showed comparable estimates of increased risk of death supporting the primary results.

Kidney failure without RRT was not a ‘clear’ or ‘absolute’ contraindication for HES according to the steering committee and scientific advisors of the trial, B Braun Melsungen AG and the Medicines Agencies in Denmark, Norway, Finland and Iceland, who all approved the protocol and the inclusion of these patients. It is important to note that the increased risk of death with HES was independent of kidney failure at inclusion in the pre-planned subgroup analysis [10].

Data on mechanical ventilation and hospital length of stay can be found in the main publication of the 6S trial as days alive without ventilation and days alive and out of hospital, respectively. We find these endpoints more valid as they are less affected by survival bias.

Taken together, the 6S trial showed that HES was harmful compared to Ringer’s acetate in patients with severe sepsis when used according to clinical practice and in alignment with the recommendations by the manufactures and authorities. Several hypotheses may be made regarding less harmful ways of using HES in critically ill patients, but clinicians, guideline committee members and authorities need to acknowledge that such safer ways have not yet been identified.

## Abbreviations

6S: Scandinavian Starch for Severe Sepsis / Septic Shock; CVP: Central venous pressure; HES: Hydroxyethyl starch; RRT: Renal replacement therapy; ScvO2: Central venous oxygen saturation.

## Competing interests

AP was the sponsor-investigator of the 6S trial, and NH was member of the Steering Committee. The 6S trial was funded by the Danish Research Council, the Rigshospitalet Research Council, and the Scandinavian Society of Anaesthesiology and Intensive Care Medicine (the ACTA Foundation). B Braun Medical delivered trial fluid to all sites free of charge. Neither the funders nor B Braun Medical had an influence on the protocol, trial conduct, data analyses or reporting of the 6S trial. AP is head of research in his department, which receives research funds from Fresenius Kabi, Germany; Cosmed, Italy and BioPorto Diagnostics, Denmark. B Braun Medical has covered his travel expenses for presenting 6S trial data at the German Anaesthetic Congress 2012 and he has received honoraria from Ferring and LFB.

RM declares that he has no competing interests.

## Authors’ contributions

NH drafted the manuscript. AP and RM contributed to the final version of the manuscript. All authors approved the final manuscript.
